# Metabolomics profiling of plasma, urine and saliva after short term training in young professional football players in Saudi Arabia

**DOI:** 10.1038/s41598-020-75755-6

**Published:** 2020-11-12

**Authors:** Mansour A. Alzharani, Ghareeb O. Alshuwaier, Khalid S. Aljaloud, Naser F. Al-Tannak, David G. Watson

**Affiliations:** 1 Poison Control and Forensic Chemistry Center, Ministry of Health, P.O. Box 42351, Medina, Kingdom of Saudi Arabia; 2 Department of Exercise Physiology, College of Sport Sciences and Physical Activity, King Saud University, Building 69, P.O. Box 1949, 11441 Riyadh, Kingdom of Saudi Arabia; 3 Department of Pharmaceutical Chemistry, Faculty of Pharmacy, Kuwait University, Kuwait City, Kuwait; 4 Strathclyde Institute of Pharmacy and Biomedical Sciences, University of Strathclyde, 161, Cathedral Street, Glasgow, G4 0RE 1 Scotland

**Keywords:** Biochemistry, Metabolomics

## Abstract

Metabolomics profiling was carried out to observe the effect of short-term intensive physical activity on the metabolome of young Saudi professional football players. Urine, plasma and saliva were collected on 2 days pre- and post-training. An Orbitrap Exactive mass spectrometer was used to analyze the samples. A reversed-phase (RP) column was used for the analysis of non-polar plasma metabolites, and a ZIC-pHILIC column was used for the analysis of plasma, saliva and urine. mzMine was used to extract the data, and the results were modelled using Simca-P 14.1 software. There was no marked variation in the metabolite profiles between pre day 1 and 2 or between post day 1 and 2 according to principal components analysis (PCA). When orthogonal partial least squares (OPLSDA) modelling was also used, and then models could be fitted based on a total number of metabolites of 75, 16 and 32 for urine, plasma and saliva using hydrophilic interaction chromatography (HILIC) and 6 for analysis of plasma with reversed-phase (RP) chromatography respectively. The present study concludes that acylcarnitine may increase post-exercise in football players suggesting that they may burn fat rather than glucose. The levels of carnitine metabolites in plasma post-exercise could provide an important indicator of fitness.

## Introduction

Physical activity plays a key role in the well-being and keeps the body fit. However, besides health benefits, exercise training protects from many diseases without any medical intervention. It can contribute an alternative treatment for people who have suffered from diseases such as type 2 diabetes^1,2^. Nowadays, diseases among humans such as obesity, cardiovascular disease and depression may be due to unhealthy habits which include a lack of exercise^2^.

On the other hand, professional soccer players, exercise regularly or daily. Those players run various distances during daily practice or a full football match, resulting in a variation in the intensity of activity between high, moderate and low intensity, which in turn causes varying demands on the different metabolic pathways involving energy production^3^. Several personal factors could affect the variation in the intensity of physical activity, for instance, lifestyle, gender and age^4–8^.

According to the type of activity, aerobic or anaerobic respiration is going to be used as the primary means to provide ATP for muscle contraction, changes in the duration and intensity of exercise lead to changes the metabolic pathways required to produce energy^3,9^.

Maximum heart rate (%HR_max_) measurement can be used to optimize the training intensity. It would be useful to determine the impact of using this measure on metabolism^10^ and perhaps ultimately assess fitness level according to the metabolic impact of exercise.

Various metabolomics studies have been conducted to study the changes to the metabolic profile due to exercise by analyzing urine or plasma samples. These studies have also identified significant changes in metabolites after long-term physical activity in colder weather. Football players were chosen because this type of sports enhance the elevation of some acylcarnitines in urine after exercise in individuals with moderate levels of fitness, and we anticipate that highly trained football players will be better to utilize acylcarnitines as energy substrates so that they are not excreted into the urine. Moreover, the levels of carnitine metabolites in plasma post-exercise could provide an important indicator of fitness^11–13^. To the best of our knowledge, just one metabolomics study, which utilized NMR spectroscopy, evaluated the effects of short-term exercise on the metabolic variations from samples of human saliva^14^. The significant metabolic pathways which are reported to be affected by exercise across several studies include purine metabolism and fatty acid metabolism, particularly the formation of acylcarnitines^13,15^. The purine metabolite hypoxanthine has been proposed as a marker of fitness since in trained individual’s purine conservation is much more efficient. Thus, hypoxanthine levels measured in physiological fluids post-exercise do not rise as much^16^.

The current study aimed to highlight the impact of a short-controlled burst of physical activity on young professional soccer players by analyzing the metabolomic profiles from samples of their urine, plasma and saliva pre- and post-exercise on 2 consecutive days. We hypothesis that there is no variation in the metabolite profiles between pre day 1 and day 2 or between post day 1 and day 2.

## Results

Metabolomics profiling of samples was carried out by using LC–MS. Extraction of data was carried out to get the output for the metabolites according to either their exact mass (with < 3 ppm deviation) or their exact mass plus retention time matching to a standard. A sample with pooled quality control (QC) samples was injected, after every 12 samples to monitor any instrumental drift over time. A clustering of QC samples indicating the stability of the instrument throughout the run was observed following principal component analysis (PCA). QC samples were not in the centre of the plot due to the pooling being random, as seen in Figure S1. Otherwise, the four-analyses carried out of the different sample types indicated clustering of the pooled samples indicating excellent instrument stability.

The polar metabolites were obtained by analysis of all biological samples using the ZIC-pHILIC column. In contrast, non-polar metabolites were analyzed by using a reverse-phase (RP) column (ACE C4 column).

Metabolites with relative standard deviation (RSD) values > 20% within the (QC) pooled samples were excluded from the analysis. In addition, the inclusion and exclusion of metabolites were according to various values variables, which have been summarised in Table 1.

**Table 1:** Filtration processes using Microsoft Excel and SIMCA-P version 14.1.

Filtration	Plasma (C4) features	Plasma (HILIC) features	Urine (HILIC) features	Saliva (HILIC) features
Total number of features, data extracted using m/z Mine 2.14	838	887	9403	9402
Unknown features which were excluded	720	587	7346	7176
Identified Metabolites with RSD > 20 which were excluded	34	300	2057	1714
Metabolites with P value < 0.05 which were included	20	211	126	331
Biomarkers were only reported as significantly influencing the models	6	75	16	32

**Figure 1:**
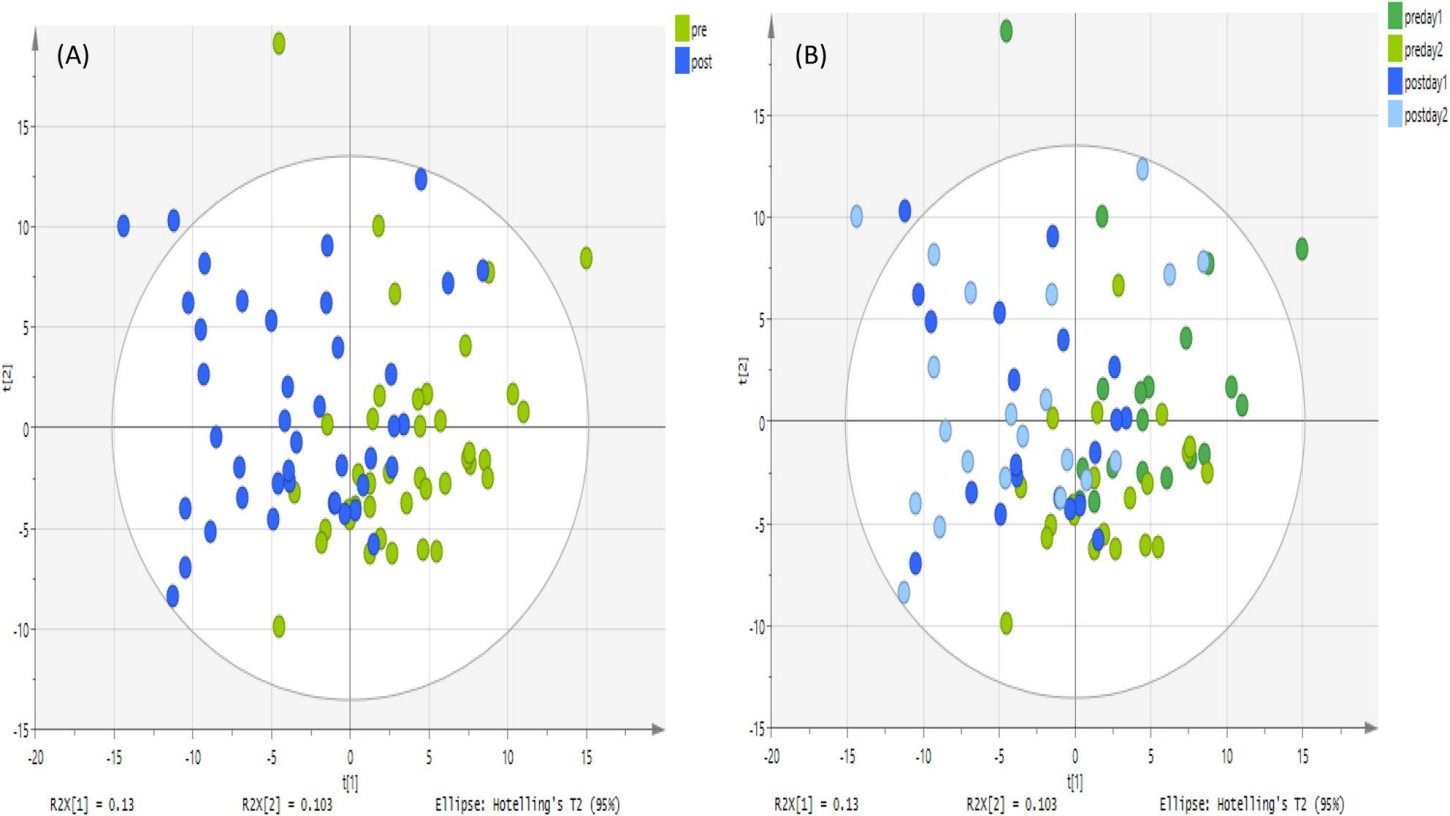
PCA score plot. It shows groups of plasma samples based on 211 metabolites analyzed on a ZIC-pHILIC column. The data was Pareto scaled. (**A**) Green is control; pre-training for both day 1 and 2 respectively (n = 40), and blue is treated; post-training for both day 1 and 2 respectively (n = 40), (**B**) green and light green are control; pre-training for both day 1 and 2 respectively, (n = 20 of each day) and blue and light blue are treated; post-training for both day 1 and 2 respectively (n = 20 for each day).

**Figure 2:**
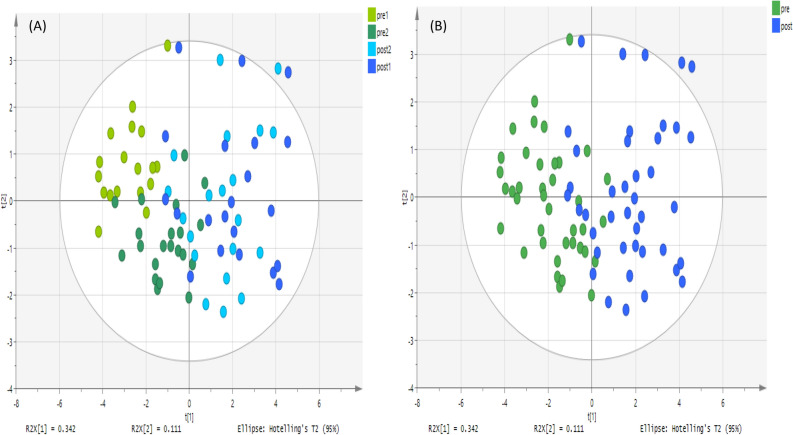
PCA score plot. It shows groups of urine samples based on 126 metabolites analyzed on a ZIC-pHILIC column. The data was Pareto scaled. (**A**) Green and light green are controls; pre-training for both day 1 and 2 respectively (n = 20 of each day). Blue and light blue are treated; post-training for both day 1 and 2 respectively (n = 20 of each day), (**B**) green is control; pre-training for both day 1 and 2 respectively, (n = 40), and blue is treated; post-training for both day 1 and 2 respectively (n = 40).

**Figure 3:**
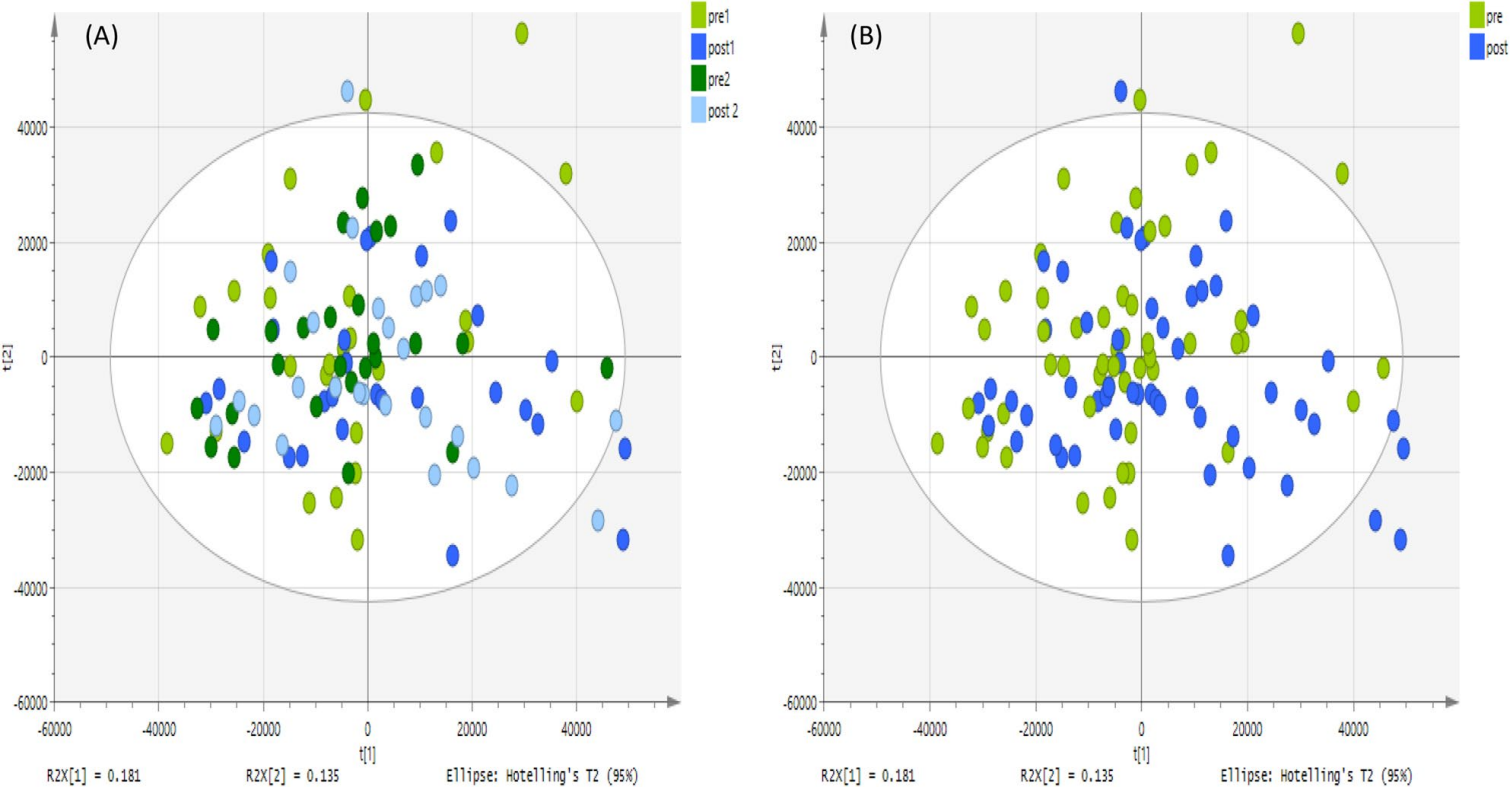
PCA score plot. It shows groups of saliva samples based on 331 putative metabolites analyzed on a ZIC-pHILIC column. The data was Pareto scaled. (**A**) Green and light green are controls; pre-training for both day 1 and 2 respectively (n = 26 of each day), and blue and light blue are treated; post-training for both day 1 and 2 respectively (n = 26 of each day), (**B**) green is control; pre-training for both day 1 and 2 respectively (n = 52) and blue is treated; post-training for both day 1 and 2 respectively (n = 52).

**Figure 4:**
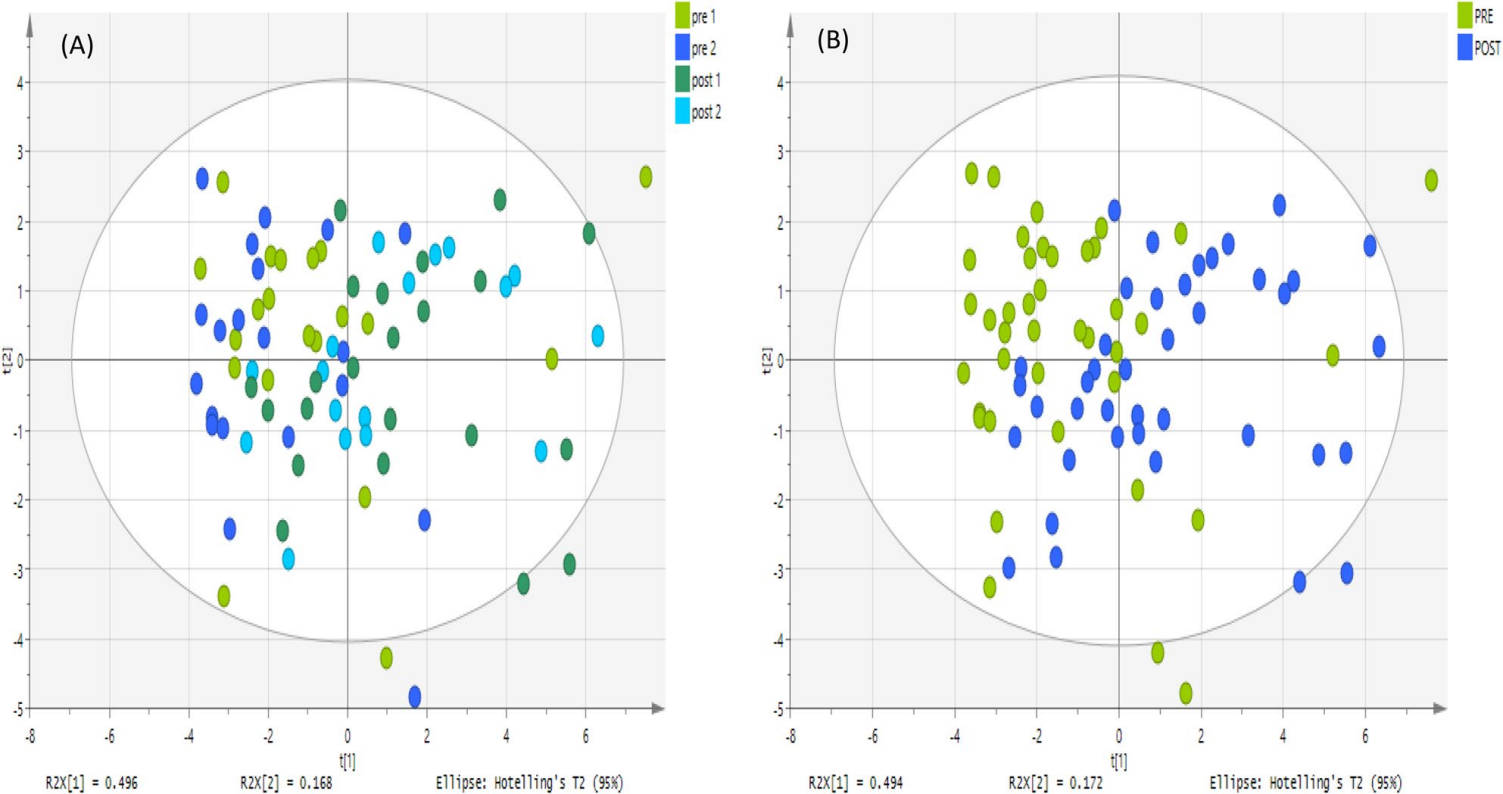
PCA score plot. It shows groups of plasma samples based on 20 metabolites analyzed on an ACE C4 column. The data was Pareto scaled. (**A**) Green and blue are control; pre-training for both day 1 and 2 respectively (n = 20 of each day), and light green and light blue are treated; post-training for both day 1 and 2 respectively (n = 20 of each day), (**B**) green is control; pre-training for both day 1 and 2 respectively (n = 40) and blue is treated; post-training for both day 1 and 2 respectively (n = 40).

Regarding the metabolites analyzed on a ZIC-pHILIC column, Figs. 1, 2 and 3 show an unsupervised classification method using PCA plots for plasma, urine and saliva samples to discover whether or not there were significant variations between pre- and post-exercise groups based on 211, 126 and 331 metabolites respectively. There is a reasonable degree of separation for plasma samples shown in Fig. 1 using the unsupervised PCA method on both days. Figure 2 shows PCA plots for the urine samples pre- and post-exercise, and in this case, the separation is more marked than for the plasma samples. The PCA plot for the saliva samples produces a less clear-cut separation between pre- and post-exercise samples in Fig. 3. PCA plots for the non-polar plasma metabolites analyzed on an ACE C4 column produced a less clear separation between pre- and post-exercise samples, as shown in Fig. 4.

**Figure 5:**
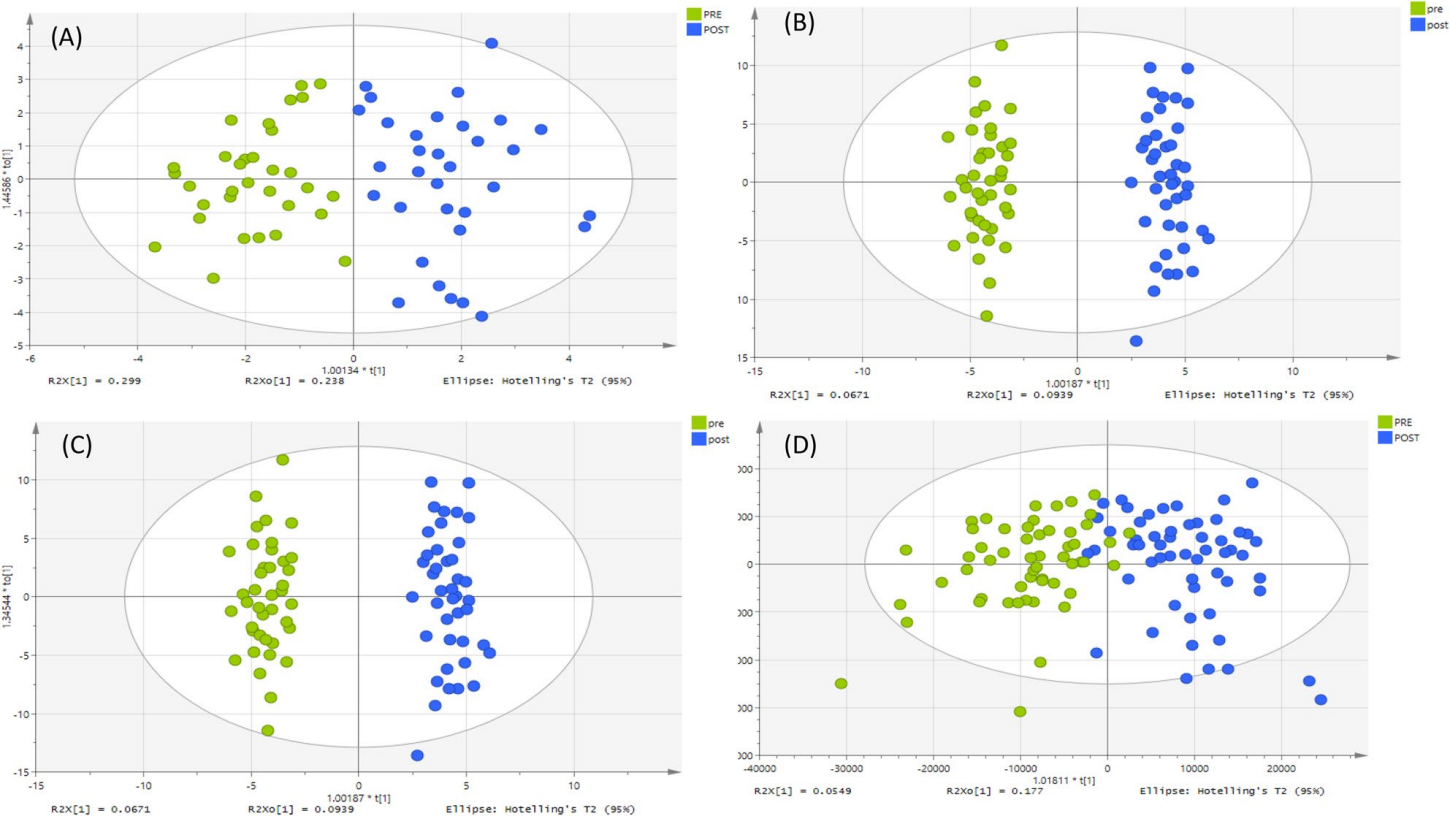
OPLS-DA score plots for day 1. The samples according to their classification; green represents the pre-training samples, while blue represents the post-training. The models were fitted based on: (**A**) 20 putative metabolites in plasma samples, analyzed on an ACE C4 column (**B**) 211 putative metabolites in plasma samples, analyzed on a ZIC-pHILIC column, (**C**) 126 putative metabolites in urine samples, analyzed on a ZIC-pHILIC column, (**D**) 331 putative metabolites in saliva samples, analyzed on a ZIC-pHILIC column, the data was Pareto scaled and log 2 transformed.

When both groups were specified, and orthogonal partial least squares discriminant analysis (OPLS-DA) was used, there was a clear separation between the pre- and post-samples in all cases (Fig. 5). Moreover, models were based on readings for 75, 16 and 32 significant polar metabolites in plasma, urine and saliva analyzed on a ZIC-pHILIC column, and 6 significant non-polar metabolites of plasma analyzed on an ACE C4 column. These metabolites were selected based on P-value < 0.05, 95% confidence interval and VIP predictive/orthogonal ratio ≥ 1 as shown in Table 2 for day 1, and Table S1 for day 2.

**Table 2:** All the metabolites affected significantly by the short-term training trial (p-value < 0.05) or fold change > 1.2 or < 0.8.

Mode	m/z	RT (min)	Putative metabolite	Urine		Plasma		Saliva	
				Ratio	p-value	Ratio	p-value	Ratio	p-value
			**Purine metabolism**						
+	136.061	8.7	Adenine			1.457	0.182	1.334	0.002
+	137.046	9.7	Hypoxanthine^a^	3.453	< 0.001	1.735	0.002	1.796	0.003
−	151.026	11.8	Xanthine			2.027	< 0.001		
−	167.021	13.3	Urate			0.766	0.009		
+	252.108	7.7	Deoxyadenosine					1.406	0.002
+	253.093	8.3	Deoxyinosine					1.713	0.037
−	283.069	13.0	Xanthosine^a^					0.136	0.001
+	298.096	6.4	5-Methylthioadenosine^a^			2.395	< 0.001		
			**Arginine and proline metabolism**						
−	104.071	13.9	4-Aminobutanoate^a^	1.394	0.004			0.661	0.019
−	112.052	9.4	Creatinine			1.278	< 0.001		
−	130.051	9.3	l-Glutamate 5-semialdehyde	0.776	0.020				
+	131.118	21.2	*N*-Acetylputrescine					1.313	0.017
+	133.097	23.5	Ornithine^a^			1.318	0.001	1.353	0.053
+	146.092	14.9	4-Guanidinobutanoate			1.603	0.014	0.585	0.017
+	148.060	15.1	Glutamate^a^			2.014	< 0.001		
+	173.104	26.1	Arginine^a^			1.521	0.009	1.577	0.013
−	176.103	15.9	l-Citrulline^a^			1.456	< 0.001		
−	188.057	9.0	*N*-Acetyl-l-glutamate			1.748	< 0.001		
+	217.129	14.6	N-acetylarginine			1.929	< 0.001		
+	247.140	13.9	*N*-(Carboxyethyl) arginine			3.494	< 0.001		
			**Pyrimidine metabolism**						
+	112.051	8.8	Cytosine			1.234	0.002	1.793	0.003
+	127.050	13.2	Thymine			1.365	0.004	1.824	0.023
+	129.066	14.6	5,6-Dihydrothymine	1.511	0.006				
+	243.097	12.4	Thymidine					2.287	0.002
			**Carnitine metabolism**						
+	204.123	10.4	Acetylcarnitine^a^			2.411	< 0.001		
+	218.138	9.1	Propionylcarnitine	0.652	0.046				
+	244.154	7.6	Tiglylcarnitine			1.549	0.012		
+	248.149	10.8	Hydroxybutyrylcarnitine			2.399	< 0.001		
	274.201	6.3	Heptanoylcarnitine			1.884	0.036		
+	276.144	12.8	Glutarylcarnitine			1.812	< 0.001		
+	290.159	12.2	Methylglutarylcarnitine			1.578	0.004		
+	302.232	5.1	Dimethylheptanoylcarnitine			1.929	0.054		
+	312.217	5.1	Decadienoylcarnitine			1.855	0.013		
+	330.227	5.1	Keto-decanoylcarnitine			1.536	0.031		
+	330.263	4.7	Dimethylnonanoylcarnitine					1.298	0.002
+	384.270	4.8	Hydroxytetradecadiencarnitine			1.411	0.041		
+	386.290	4.7	Hydroxytetradecenoylcarnitine			1.505	0.045		
			**T.C.A. cycle**						
−	133.014	16.9	Malate^a^			3.226	< 0.001		
−	145.014	15.9	2-Oxoglutarate^a^			1.954	< 0.001		
−	173.009	19.0	cis-Aconitate^a^			3.632	< 0.001		
−	191.020	19.2	Citrate^a^			2.010	< 0.001		
			**Pentose phosphate pathway**						
−	133.051	9.3	Deoxyribose			1.330	0.008		
−	149.046	11.9	Ribose^a^			1.545	< 0.001		
−	151.061	12.9	Xylitol or isomer^a^			1.328	0.008		
−	193.036	16.8	Glucuronate or isomer^a^			1.896	< 0.001		
−	195.051	15.0	Gluconic acid^a^			2.852	0.002		
−	229.011	15.7	Ribose 5-phosphate^a^					0.145	0.05
			**Glycine, serine and threonine metabolism**						
+	76.076	10.5	Aminopropan-2-ol			2.002	0.018		
−	105.019	12.6	Glycerate			1.665	0.001		
+	118.061	15.8	Guanidinoacetate	1.604	0.028				
+	120.065	11.3	Threonine^a^			1.586	0.015		
			**Lysine metabolism**						
−	126.056	7.2	2,3,4,5-Tetrahydropyridine-2-carboxylate	1.154	0.021	1.614	0.001		
+	146.081	14.6	Hexanoic acid			1.364	0.048	0.232	0.024
+	146.117	12.9	Trimethylammoniobutanoate			1.237	0.001		
+	147.113	23.6	l-Lysine^a^			1.611	0.02		
+	160.097	6.9	Acetamidopentanoate	1.151	0.028				
+	162.112	13.3	l-Carnitine^a^			3.264	0.011		
+	189.159	22.1	N6,N6,N6-Trimethyl-l-lysine			1.859	< 0.001		
+	204.086	14.2	N2-Acetyl-l-aminoadipate			1.532	< 0.001		
+	205.118	10.3	N6-Acetyl-N6-hydroxy-l-lysine			2.175	< 0.001		
+	219.133	14.4	Carboxyethyllysine					1.465	0.017
			**Histidine metabolism**						
+	137.036	7.6	Urocanate^a^			1.733	< 0.001		
+	141.066	9.7	Methylimidazoleacetic acid			1.632	0.010		
+	170.092	12.8	Methylhistidine					0.629	0.029
			**Tryptophan metabolism**						
+	118.065	10.1	Indole^a^			1.506	0.028	1.596	0.003
+	161.107	10.1	Tryptamine					1.912	0.003
+	177.102	13.4	Serotonin					1.362	0.056
−	204.067	8.1	Indolelactate			1.394	< 0.001	1.352	0.058
−	219.077	8.9	5-Hydroxy-l-tryptophan isomer					2.043	< 0.001
			**Tyrosine metabolism**						
+	138.091	5.7	Tyramine^a^					1.212	0.006
−	179.035	8.3	Hydroxyphenylpyruvate			1.606	< 0.001		
−	181.051	9.5	Hydroxyphenyllactate			1.782	< 0.001		
+	182.081	12.3	l-Tyrosine^a^	0.802	0.056			1.333	0.055
			**Valine, leucine and isoleucine degradation**						
−	115.04	4.8	3-Methyl-2-oxobutanoic acid			0.752	0.002		
−	129.056	4.3	Methyl-oxopentanoic acid			0.761	< 0.001		
+	132.101	10.8	l-Leucine					1.669	< 0.001
			**Phenylalanine metabolism**						
+	122.096	4.7	Phenethylamine					1.322	0.004
+	123.044	13.3	Benzoate					1.396	0.047
+	136.075	13.3	Phenylacetamide					1.387	0.021
+	149.059	10.1	Cinnamate					1.585	0.003
−	166.086	10.1	Phenylalanine^a^					1.606	0.004
			**Methionine metabolism**						
+	150.058	11.3	l-Methionine^a^	1.404	0.053				
+	166.053	13.4	Methionine S-oxide			1.628	< 0.001		
−	176.039	7.2	*N*-Formyl-l-methionine			1.241	0.004		
+	192.105	4.9	Trihomomethionine			1.408	0.010		
			**Alanine and aspartate metabolism**						
−	88.04	14.6	l-Alanine^a^			1.439	0.002		
−	225.099	11.7	Carnosine^a^					2.051	0.007
			**Fatty acids and metabolites**^**C4**^						
−	131.071	2.0	Hydroxyhexanoic acid^a,b^			1.526	< 0.001		
−	227.202	15.9	Tetradecanoic acid^b^			1.498	0.015		
−	241.218	17.4	Pentadecanoic acid^a,b^			1.208	0.035		
−	253.218	16.8	Hexadecenoic acid^b^			1.374	0.047		
−	255.233	18.8	Hexadecanoic acid isomer^b^			1.233	0.054		
−	281.249	19.5	Octadecenoic acid^b^			1.366	0.032		
			**Miscellaneous**						
−	147.03	15.8	(R)-2-Hydroxyglutarate			0.674	0.006		
+	241.031	16.5	L-Cystine^a^	1.041	0.041				
−	259.022	15.8	D-Glucose 1-phosphate^a^			1.304	< 0.001		
+	345.139	12.3	Melibiitol			0.337	0.003		
−	448.307	5.2	Glycodeoxycholate			0.196	< 0.001		
−	464.302	5.5	Glycocholate^a^			1.278	< 0.001		

^a^Matches retention time of standard.

^b^Data from runs on ACE C4 column, otherwise run on the pHILIC column.

The OPLSDA plots clearly show significant separation between the two groups with P CV-ANOVA = 7.81E − 29, 1.17E − 027 and 3.11E − 020 of plasma, urine, saliva analyzed on a ZIC-pHILIC column respectively, where P CV-ANOVA = 7.74E − 012 for plasma analyzed on an ACE C4 column. The validity of these supervised models was assessed based on the permutations test plot and cross-validation. In the permutation test plots, R2 and Q2 parameters obtained from the original model are compared to newly permuted R2 and Q2 values, and to confirm the validity using this test. The new parameters generated from this permutation should all be lower in value than the original values as well as the regression line of the predictive model should cross the horizontal below zero line see Figures S2, S4, S6 and S8. The Observed vs Predicted plot of all models were plotted are shown in Figures S3, S5, S7 and S9. These plots display the observed versus the predicted value of the selected Y-variable. Also, the regression line R2 is close to one, which indicates an excellent and valid model. However, the regression line in the plots were R2 = 0.95, 0.95 and 0.85 for plasma, urine and saliva respectively, analyzed on a ZIC-pHILIC column, and R2 = 0. 77 was obtained for plasma analyzed on an ACE C4 column, indicating the validity of the cross-validation of the model.

In Table 2, the univariate comparisons of the changes in the metabolites from pre- and post-exercise for day 1 samples are shown, while those for day 2 are shown in Table S1. Hypoxanthine was the only polar metabolite, which was found to change in all cases (plasma, urine and saliva) on day 1 where it consistently increases. However, on day 2, it did not change consistently. Otherwise, many significant differences in metabolites were obtained from plasma; the levels of polar and non-polar (lipophilic) metabolites of plasma analyzed on both columns were increased in most cases. Moreover, the levels of polar metabolites obtained from urine and saliva were also found to be slightly increased in many cases compared to the pre-training levels.

## Discussion

The intensity of the physical activity plays an important role in changing metabolic profile as observed in previous studies where two cohorts were compared, e.g., pre- and post-training^11–13,17^. In the current study, determination of the heart rate level was used to indicate the effect of physical activity on metabolism since the body uses different substrates to produce energy according to the intensity of exercise and heart rate measurement. The heart rates were 70% and 72% of the maximum on the first and second days respectively. These percentages indicate that the training could be defined as moderate-intensity exercise according to participants ages, which may explain the metabolite ratios before and after-training^10^. In general, two measurements have been used in exercise studies, maximum heart rate (%HR_max_) and oxygen uptake (VO_2max_). The latter measurement can be useful for measuring high-intensity exercise because the oxygen uptake under anaerobic respiration or maximum load increases more relative to VO_2max_ than to %HR_max_^10^.

Adenosine triphosphate (ATP) is the primary source of energy in the body, which is used by cells for muscle movements or biosynthesis^18^. Therefore, it is essential first to understand the process for generating ATP to produce energy in the body within the rest and active situations, and this understanding is leading us to realize the interaction of the effects on metabolism according to the intensity of exercise.

ATP function is to allow muscle contraction and active fuel transport pumps^19^. The last phosphate in the triphosphate structure is an unstable bond, which can be broken through a hydrolysis reaction; when H_2_O is introduced with the assistance of the ATPase enzyme to form adenosine diphosphate (ADP). ATP is then converted to ADP with the release of energy; this energy is utilized by the cell for functions such as movement driven by conformational changes in proteins. Therefore, because cells need ATP always for energy, ATP must be formed from ADP by creatine phosphate to form creatine and ATP with the assistance of the creatine kinase enzyme. ADP is converted to ATP via an endothermic process that requires energy; that comes from cellular respiration (aerobic); this cycle is called the ATP cycle^9^. Consequently, when muscles are being active or contract through exercise, the production of ATP occurs in three steps depending on the intensity of exercise. First, creatine phosphate transfers its phosphate group back to ADP to form creatine and ATP as a high-energy phosphate, which then can be used to create muscle contraction, this ATP cycle runs out very quickly and is not sufficient for a long duration of exercise^9^. Secondly, since creatine phosphate is depleted quickly within a short period, the muscles then turn to the glycolysis process as a second source for producing ATP. Glucose is broken down to generate ATP but not as quickly as via creatine phosphate generation due to the length of the process of glycolysis. Glucose can diffuse straight into the muscle from the blood or can be formed from glycogen. Glycogen is a polysaccharide, which is stored in the muscle tissue after conversion from glucose. Glycogen must first be converted to glucose by the glycogenolysis^9^. The glucose resulting from both processes undergoes glycolysis, which is a series of reactions that ultimately produces two ATP molecules and pyruvate. Pyruvate or pyruvic acid can be used for two things. It can be broken down into lactic acid by reaction with the reduced form of nicotinamide adenine dinucleotide (NADH), catalyzed by lactate dehydrogenase. Therefore, pyruvate gains electrons to become lactate and NADH is oxidized to form NAD+. The NAD+ molecule allows glycolysis to keep going. This process is called anaerobic respiration. This process is not efficient because it only makes 2ATP molecules per glucose molecule, which is ultimately going to be depleted when the intensity of exercise is high for a short time^9^.

The third source for generating ATP; that is good for a long duration, is called aerobic cellular respiration. In the Krebs or citric acid cycle (TCA cycle), pyruvate can be used to produce NADH. The NADH produced enters the electron transport system of the terminal respiratory chain. It is used to create ATP with the protons from NADH, in the end, being transferred to molecular oxygen. The outcome again is the formation of CO_2_, H_2_O and ATP. This process is efficient because it makes 36 ATP molecules per glucose molecule and 100 molecules of ATP per fatty acid^9^. This process needs a steady supply of oxygen, which makes it much slower than glycolysis. This process can be understood in terms of stress since aerobic exercise burns fat at low-intensity exercise and stress. In contrast, the high-intensity anaerobic exercise burns sugar as the primary fuel. The body needs to control this high stress by releasing a stress hormone called cortisol to raise the blood sugar level, and as a consequence, produces lactic acid^9^.

Firstly, glucose is broken down by glycolysis to produce pyruvate that is used first for aerobic respiration. Secondly, when glucose is depleted, then the body turns to break down fat to form fatty acids and glycerol to participate in the aerobic process. Finally, when protein is high or fat, and glucose stores are deficient, then protein can break down to form amino acids to be used in this process^9^. This catabolism occurs within the mitochondria, the powerhouse of muscle cells, in the presence of oxygen that is from either hemoglobin in the blood or oxygen attached to myoglobin in the muscles.

By looking at the training design in this project and the significantly changed metabolites that are summarised in Tables 1 and Table S1, and compared that with previous studies, then the output of metabolomics changes could be modified according to the style of exercise^11,12,17^. In the current case, it was found that the majority of the polar and non-polar metabolites detected were increased slightly after training due to the type of exercise.

Purine metabolites are considered a leading indicator of the effects of exercise, specifically hypoxanthine^17^. Activation of this pathway requires high physical activity. Hypoxanthine was increased by more than half in plasma and saliva and more than three times in urine, as well as xanthine the oxidation product of hypoxanthine, the concentration of which doubled and was observed just in plasma. The possible justification is that hypoxanthine is converted to xanthine by xanthine dehydrogenase; that is present in liver and the intestine, where hypoxanthine flows out of the bloodstream and saliva and is therefore present highly in the urine^20^. Elevation of hypoxanthine post-exercise samples was observed in previous studies and is increased in high-intensity training as well as being higher in urine than in plasma^11,12^. However, on day 2 of exercise, hypoxanthine fell the following exercise and this suggesting that purine conservation increases with the repetition of training. Adenine was found to be increased in plasma and saliva. However, since it is re-absorbed by the kidney, this may explain its absence in urine^9^.

The carnitine pathway was the most obviously affected after training and produced the highest number of significantly changed metabolites in plasma. This impact of exercise on the carnitine metabolites could be due to mitochondrial fatty acid oxidation since acetylcarnitine accumulation has been identified to be high in plasma following exercise, which indicates an increased demand for stored energy^15^. Acylcarnitines could also play an essential role in the regulation exertion when interacting with the neurons regulating muscle activity^15^. Acetyl carnitine was consistently elevated in plasma on both training days; previous research has found that carnitine acetyltransferase, which transfers the acetyl group from acetyl CoA to carnitine forming acetylcarnitine, was important for maintaining muscle performance^21^. The acetylcarnitine might function as a source of acetate for the rapid formation of acetyl CoA when it is depleted. It is important to note that acylcarnitines can be directly converted to acyl CoAs without the requirement for the investment of ATP, which is required to convert fatty acids to their CoAs. There are many other elevated acylcarnitines in plasma post-exercise on both days, and these may derive from peroxisomal metabolism of fatty acids^11^. The fact that the acylcarnitines are elevated in plasma but not in urine suggests that they are not waste products but maybe critical metabolic substrates for muscle activity. In an earlier paper, we observed an elevation of some acylcarnitines in urine after exercise^12^ in individuals with moderate levels of fitness, and it may be that in the current study the highly trained football players are better able to utilize acylcarnitines as energy substrates so that they are not excreted into the urine.

The metabolic pathway of arginine-proline appears to have high numbers of metabolites which are significantly increased in plasma samples, and it was found that ornithine and arginine were changed dramatically in plasma and saliva samples. This change was more marked on training day 1. Also, glutamate, the precursor of arginine was increased in the plasma samples. It has been found that glutamate can be an essential source for cell energy metabolism, where it is involved in brain energy metabolism and neuronal functions and survival^22^. N-(Carboxyethyl) arginine; which is formed from arginine, was found to be increased three-fold after training. This compound is formed via the reaction between arginine and methylglyoxal, which is formed during glycolysis, and it has been suggested that it is an inhibitor of nitric oxide action. Also, arginine increased by 50%, which can serve as a precursor of various amino acid substances such as glutamine, citrulline, proline and creatine, which are also shown to change with exercise^23,24^. The conversion arginine to citrulline, which is elevated in plasma following exercise, produces nitric oxide, which produces vasodilation^9^.

Several metabolites in the lysine metabolism pathway are elevated in plasma post-exercise. Perhaps most significantly carnitine and its precursor trimethyl lysine. Several metabolites in the histidine metabolism pathway are elevated in plasma post-exercise. Urocanic acid and its metabolite imidazolone propionate are elevated. Urocanic acid is formed in the skin as a result of exposure to UV radiation, and this would be consistent with exposure to sunlight during the training session^25^. Imidazole acetic acid is a metabolite of histamine, and it has been shown that histamine levels rise in the blood post-exercise and are responsible for promoting vasodilation. Histamine was not detected directly, but increases in imidazole acetic acid may indicate an increase in its release and subsequent metabolism.

TCA and some fatty acids showed higher responses in samples post-training; the change was observed only in the plasma samples. Once fatty acid metabolites were increased, due to hydrolysis of triglycerides to fatty acids and glycerol. The fatty acids can be broken down during the beta-oxidation process to acetyl-CoA, which used in the citric acid cycle (TCA). Some TCA metabolites were increased. It has been proposed that increased fat oxidation could lead to a reduced risk of cardiovascular disease (CVD)^26^. It has been reported that increases in the concentration of malate lead to increased rates of oxidation of succinate and citrate and it seems to be the indicate an increased rate of oxidation of fatty acids^24^. In the present work, malate was increased significantly in plasma; three-fold post-training in comparison to pre-training, which led to an increase in the succinate and citrate levels. Citrate was elevated two-fold after training, and citrate elevation post-exercise has been observed previously^27^. It has been shown that citrate can inhibit glycolysis via inhibition of fructose kinase^28^ thus diverting energy metabolism towards fatty acid oxidation. The effects on levels of TCA metabolites were less marked on day 2.

## Conclusions

This study considered to be the first study using an untargeted metabolomics approach of plasma, urine and saliva samples to determine the metabolic profile in response to short-term training in young professional football players. Although there are many metabolites, which were affected by this type of exercise, the majority of metabolites were found to be increased. However, the increase in the metabolites were not very high in comparison to other studies^12,17^ that it might be due to the short duration and calibrated intensity of the exercise in combination with the fitness of the participants. Our study found out that there were a significant increase obsererved in acylcarnitines, which are involved in fatty acid oxidation, post-exercise on 2 exercise days, which suggests that these highly trained individuals burn fat rather than glucose. Moreover, purine metabolites were slightly increased, especially hypoxanthine and its product xanthine. Furthermore, the levels of carnitine metabolites in plasma post-exercise could provide an important indicator of fitness. Recently, the decrease in the activity of the carnitine shuttle have been associated with increased frailty in old age^29^. Inconclusion, although metabolic changes can be observed post-exercise in saliva and urine, plasma gives a more comprehensive picture.

## Materials and methods

### Ethical statement

Collection of urine, blood and saliva samples was approved by the ethical committee at King Saud University. All sampling handling was carried out in accordance with relevant guidelines and regulations, and as approved by the above authorities. Written informed consent from all the study participants were obtained.

### Chemicals and solvents

HPLC grade acetonitrile (ACN) was purchased from Fisher Scientific (Loughborough, UK). HPLC grade water was produced by a Direct-Q 3 Ultrapure Water System from Millipore, U.K. AnalaR-grade formic acid (98%) was obtained from BDH-Merck (Poole, UK). Ammonium carbonate was purchased from Sigma-Aldrich (Poole, UK). Authentic stock standards were prepared as stated previously in the literature and diluted four times with ACN before LC–MS analysis, then distributed into seven different standard solutions^30^. ^13^C_2_ glycine was provided Sigma-Aldrich (Poole, UK). A mixture of fatty acid standards was prepared from a mix of 37 fatty acid methyl ester standards were supplied by Sigma Aldrich (Supelco 37 component FAME Mix) by hydrolysis with 1 M methanolic KOH.

### Participants

Twenty-six professional soccer players (age 20.6 ± 1.4 years, body mass 70.2 ± 1.6 kg, height 178 ± 2 cm and BMI 22.2 ± 0.5 kg/m^2^ participated in the study. All players attended the training sessions during the mid-season in February 2018. Players train for 5 days, 90 min per day and play one match per week. The 2 days on which the collection of samples performed was during the training sessions on 2 consecutive days. No participants were injured, and no participants took any medication during the study. The participants attended the club one and a half hours before the commencement of training every day. The Ethics Committee at King Saud University approved the study, and the consent for study participation was obtained from all participants; all players read the information sheet and signed a consent form before participating in the study. Seven of players are members of the National team for players under 23 years old.

Plasma and urine samples were collected from 20 soccer players. While 18 participants out of the 20 who donated urine and plasma samples, also donated saliva samples; eight additional soccer players only donated saliva samples to make a total of 26 saliva samples. Written informed consent from all the study participants were obtained.

### Experimental design

The collection of plasma, urine and saliva samples from young Saudi professional soccer players was conducted in Riyadh, Saudi Arabia by King Saud University, College of Sport Sciences and Physical Activity, Exercise Physiology Department. Samples were stored at − 80 °C in the laboratory. Samples were shipped in dry ice and took 2 days to arrive for analysis at the University of Strathclyde, UK.

The collection of samples was done on 2 consecutive days, pre- and post-training sessions. The total number of samples were, 80 plasma and urine samples each, as well as 104 samples of saliva. The training sessions took roughly 2 h. All training sessions were started at the same time each day. Two training sessions were designed for the study, which began with a 15 min warm-up. The players were then divided randomly into groups to play two small side games for 30 min, followed by a 4 min rest period. The coach also set up a game for 40 min divided into two halves, followed by a 5–10 min cool down. The intensity of the training on the first and the second training day was designed based on the percentage of maximum heart rate. The heart rate averages were 70% and 72% of the maximum on the first and second days respectively. The mean ambient temperature was 25 ± 3 °C, and humidity was 18 ± 4% for the first day and 26 ± 1 °C and 16 ± 2% for the second day.

Individual containers in sealed coded bags were given to the players for the pre- and post-training samples.

On the days of collection, the methods used for collecting samples were explained to the participants. As a part of the collection procedure, volunteers placed their labelled samples directly into the fridge until the end of the training sessions. Subsequently, all samples were placed in environmental storage and transported immediately to the laboratory at the College of Sport Sciences and Physical Activity for proper storage.

### Samples collection

For the collection of urine samples, soccer players were required to clean their hands and penis first, as well as collect midstream urine during a full flow of urine to avoid any contamination. Samples were collected in a universal 30 ml container and placed in a small sealed plastic labelled bag and left it in the fridge. Finally, to empty their bladder during per training sample collection. Individual urine samples were transferred in aliquots of 3 ml into Eppendorf tubes before being stored at − 80 °C.

For the collection of plasma samples, whole blood samples were collected from veins into a 10 ml EDTA tube that contained an anticoagulant (BD Vacutainer Systems, Plymouth, UK). All samples were placed on ice and delivered immediately to the laboratory. All blood tubes were centrifuged immediately at 1500 × *g* for 15 min at 4 °C. Aliquots of 3 ml of the plasma samples were then transferred into Eppendorf tubes before being stored at − 80 °C.

For the collection of saliva samples, a Sarstedt Salivette polyester tube was used. The sampling procedure was carried out as follows: after hand washing, the wad is taken into the mouth, and left for 2 min and then returned to tube. The volunteers were asked not to brush their teeth within 1 h of collecting the samples. They are also required not to have any food or drink within 30 min of taking a sample to avoid any contamination or interference matrix. All samples were placed on ice and delivered immediately to the laboratory. According to the manufacturer’s instructions, samples were centrifuged at 1000 × *g* for 20 min at 4 °C; then the inlay was removed from tubes and samples were then stored at − 80 °C.

### Samples preparation

Samples stored at − 80 °C were allowed to thaw and equilibrate to room temperature for 1–2 h before further use. Metabolites were extracted by transferring 200 μL of samples to an Eppendorf tube followed by the addition of 800 μL of acetonitrile (ACN) containing 5 µg/mL of ^13^C_2_ glycine as an internal standard to ensure retention time stability and then vortexed. The samples were then centrifuged at 8000 revolutions per minute for 10 min. The supernatant was then collected into an HPLC vial as a final solution ready for LC–MS analysis. Samples were organized into batches corresponding to the player donor. Each group injected together as follows B = pre-day 1, R = post-day 1, G = pre-day 2 and K = post-day 2, for example, B1, R1, G1 and K1, were samples collected from player 1 in pre- and post-training for the 2 days of training.

The accuracy, as well as reproducibility of the analytical method, were measured by regularly injecting authentic standard metabolite mixtures and quality control (QC) samples throughout the runs. The analytical standards were prepared by adding 10 μg/mL as the final concentration of each metabolite standard plus ^13^C_2_ glycine, into the seven different standard solutions^30^. The pooled quality control (QC) samples were prepared by pipetting 10 µL from random groups and then mixing them. A mixture of fatty acid standards was prepared from a mix of 37 fatty acid methyl ester standards supplied by Sigma Aldrich (Supelco 37 component FAME Mix) by hydrolysis with 1 M methanolic KOH followed by extraction into hexane.

### LC–MS conditions

An Accela HPLC system interfaced to an Exactive Orbitrap mass spectrometer (Thermo Fisher Scientific, Bremen, Germany) was used for the liquid chromatographic separations. ZIC-pHILIC (150 × 4.6 mm, 5 µm) and ACE C4 (150 × 3.0 mm, 3 µm) HPLC columns supplied by HiChrom (Reading, UK) were used, since the former column was used for all biological samples, whereas, latter was used just for plasma samples. Samples were run on LC–MS under the following conditions: the ZIC-pHILIC mobile phase consisted of 20 mM ammonium carbonate in HPLC-grade water (A) and acetonitrile (B). The solvent gradient used was 80% B (0 min), 20% (30 min), 8% (31–36 min), and 80% (37–45 min) at a flow rate of 0.3 mL/min. For the ACE C4 column, the mobile phase was 1 mM acetic acid in water (A) and 1 mM acetic acid in acetonitrile (B). The solvent gradient used was 40% B (0 min), 100% (30–36 min) and 40% (37–41 min) at a flow rate of 0.4 mL/min. The nitrogen sheath and auxiliary gas flow rates were maintained at 50 and 17 arbitrary units. The electrospray ionization (ESI) interface was employed in a positive/negative dual polarity mode, with a spray voltage of 4.5 kV for positive mode and 4.0 kV for negative mode, while the ion transfer capillary temperature was set at 275 °C. Full scan data were obtained in the mass-to-charge ratio (m/z) between 75 and 1200 amu for both ionization modes. The data were collected and processed using Xcalibur 2.1.0 software (Thermo Fisher Scientific, Bremen, Germany).

### LC–MS data processing with m/z mine and statistical analysis

M/z mine 2.14 was used as processing for raw LC–MS files^31^. The Metlin database, the Lipid Maps and Human Metabolome DataBase HMDB web (https://www.hmdb.ca/) were used to prepare an in-house metabolite database to investigate metabolites and identify the accurate masses. All reported metabolites were within 3 ppm of their exact masses.

Univariate and Multivariate data analysis were employed. Former analysis achieved using Microsoft Excel 2016 and paired t-tests between pre and post samples and differences were considered significant at p < 0.05. Whereas latter analysis performed using SIMCA-P software v.14.0 (Umetrics, Umea, Sweden), which included analysis (PCA-X) and (OPLS-DA).

The sample size was calculated using G-Power program (version 3.1.9.7). The calculation revealed that 27 participants were required to detect a moderate effect size (0.5) in the metabolite profiles with a power of 80% and at > 0.05 significance level. This calculation was based on data reported in a previous study^16^. Therefore, 30 participants were aimed to be recruited to accommodate an expected maximal dropout rate of 10%. However, the total number of the participants who completed all parts of the study was 26 football players.

## Supplementary information

Supplementary Information.
