# Supplementary material for: Metabolomics profiling of plasma, urine and saliva after short term training in young professional football players in Saudi Arabia
Source: Sci Rep. 2020 Nov 12;10:19759. doi: 10.1038/s41598-020-75755-6 (PMC7665217; doi:10.1038/s41598-020-75755-6)
Supplement: Supplementary file 1 — Supplementary Information. [file 41598_2020_75755_MOESM1_ESM.docx]

Article

Metabolomics Profiling of Plasma, Urine and Saliva After Short Term Training in Young Professional Football Players in Saudi Arabia

Mansour A. Alzharani ^1 ,^* , Ghareeb O. Alshuwaier ^2^, Khalid S. Aljaloud ^2^ , Naser F. Al-Tannak^3.*^ and David G. Watson ^4,^

^1^ Poison Control and Forensic Chemistry Center, Ministry of Health, P.O. Box 42351, Medina KSA; M.A.A. [mansour-1399@hotmail.com](mailto:mansour-1399@hotmail.com).

^2^ College of Sports Sciences and Physical Activity, King Saud University, Building 69, Code 11441, P.O. Box 1949, Riyadh KSA; G.O.A.[galshuwaier@KSU.EDU.SA](mailto:galshuwaier@KSU.EDU.SA); K.S.A. [Khaljaloud@KSU.EDU.SA](mailto:Khaljaloud@KSU.EDU.SA).

^3^ Department of pharmaceutical chemistry, Faculty of pharmacy, Kuwait University. [Dr.altannak@ku.edu.kw](mailto:Dr.altannak@hsc.edu.kw)

^4^ Strathclyde Institute of Pharmacy and Biomedical Sciences, 161, Cathedral Street, University of Strathclyde, Glasgow G4 0RE 1; D.G.W. [d.g.watson@strath.ac.uk](mailto:d.g.watson@strath.ac.uk).

***** Correspondence: [mansour-1399@hotmail.com](mailto:mansour-1399@hotmail.com) and [Dr.altannak@ku.edu.kw](mailto:Dr.altannak@hsc.edu.kw)

Supplemental material content:

**Supplementary Figure 1.** PCA scores plots for QC (pooled) extract samples.

**Supplementary Figure 2**. Permutations test of plasma analysed on a C4 column.

**Supplementary Figure 3**. The observed versus predicted test of plasma analysed on a C4 column.

**Supplementary Figure 4**. Permutations test of plasma analysed on a ZIC-pHILIC column.

**Supplementary Figure 5**. The observed versus predicted test of plasma analysed on a ZIC-pHILIC column.

**Supplementary Figure 6**. Permutations test of urine analysed on a ZIC-pHILIC column.

**Supplementary Figure 7**. The observed versus predicted test of urine analysed on a ZIC-pHILIC column.

**Supplementary Figure 8**. Permutations test of saliva analysed on a ZIC-pHILIC column.

**Supplementary Figure 9**. The observed versus predicted test of saliva analysed on a ZIC-pHILIC column.

**Supplementary Table 1**. The significant metabolites effected by the short-term training trial in the day 2.


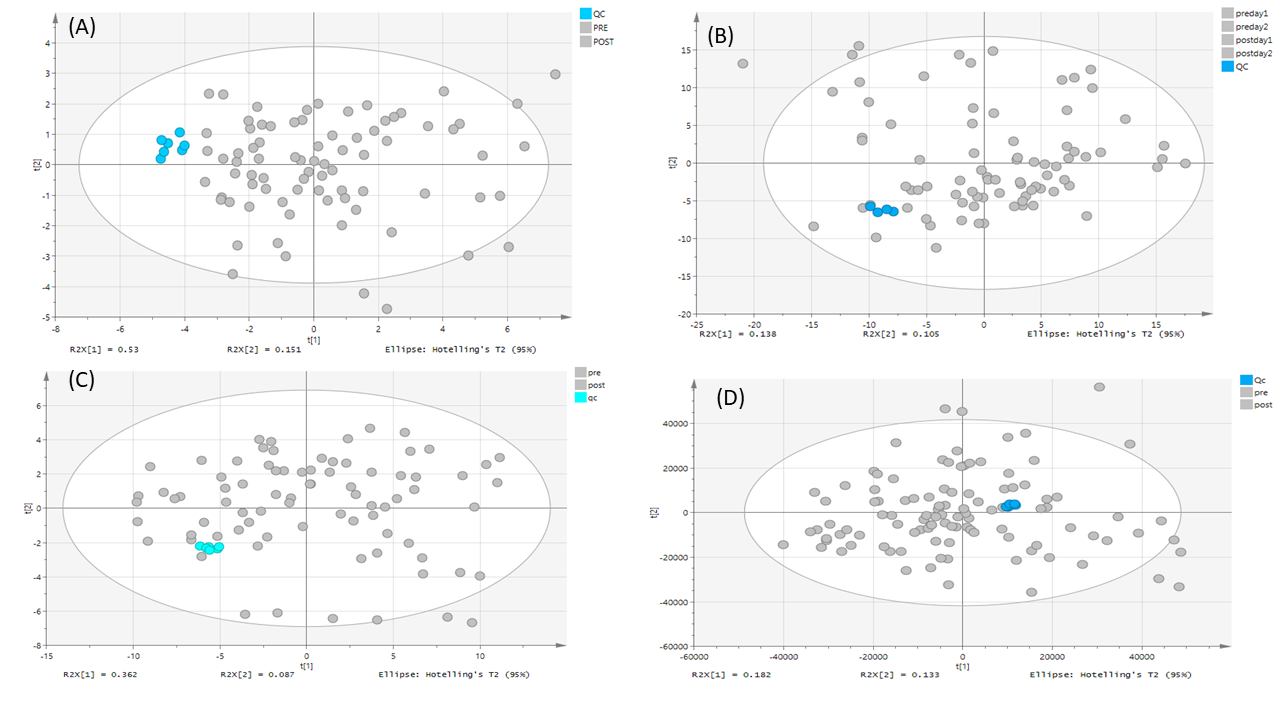


Figure S1: PCA scores plots for QC (pooled) extract samples of (A) plasma (n=7), analysed on an ACE C4 column. (B) plasma (n=6), analysed on a ZIC-pHILIC column. (C) urine (n=5), analysed on a ZIC-pHILIC column. (D) Saliva (n=6), analysed on a ZIC-pHILIC column. The plots show the clustering of pooled samples (QC) compared to the rest of the samples (grey-No class), the data was Pareto scaled.


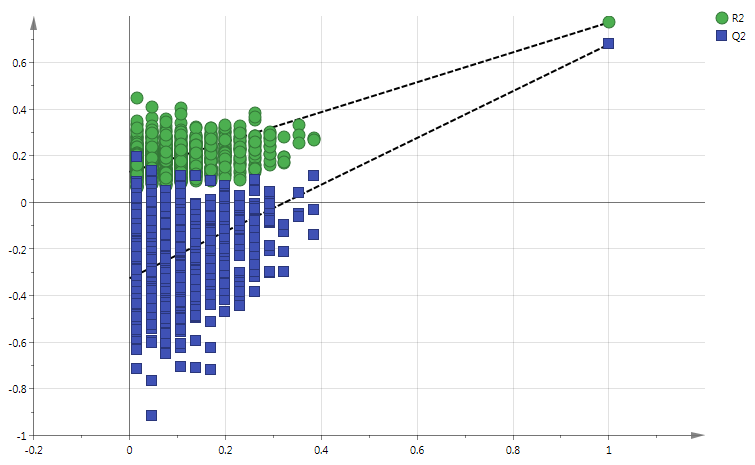


figure S2 Permutations test. The plot shows, for a selected Y-variables, on the vertical axis the values of R2 and Q2 for the original model (far to the right) and of the Y-permuted models further to the left. The horizontal axis shows the correlation between the permuted Y-vectors and the original Y-vector for the selected Y. The original Y has the correlation 1.0.


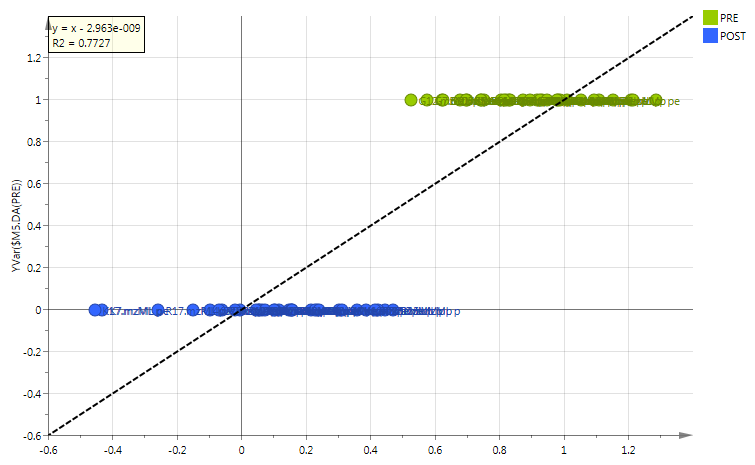


figure S3: The plot displays the observed (y-axis) versus predicted (x-axis) values of the selected Y-variable of the model. The R2 of the regression line indicates the goodness of Fit = 0.77.

| 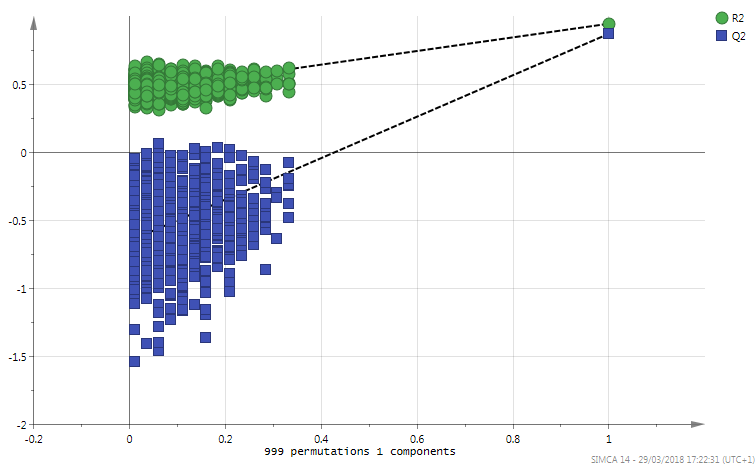  figure S4: Permutations test. The plot shows, for a selected Y-variables, on the vertical axis the values of R2 and Q2 for the original model (far to the right) and of the Y permuted models further to the left. The horizontal axis shows the correlation between the permuted Y-vectors and the original Y-vector for the selected Y. The original Y has the correlation 1.0 |
| --- |
| 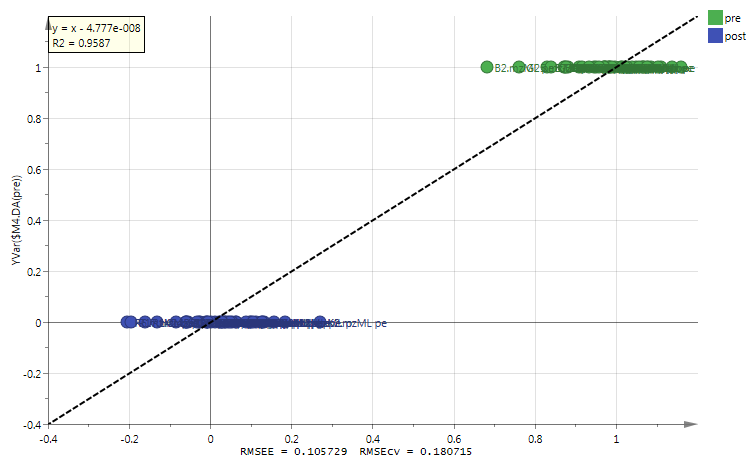  figure S5: The plot displays the observed (y-axis) versus predicted (x-axis) values of the selected Y-variable of the model. The R2 of the regression line indicates the goodness of Fit = 0.95. |

| 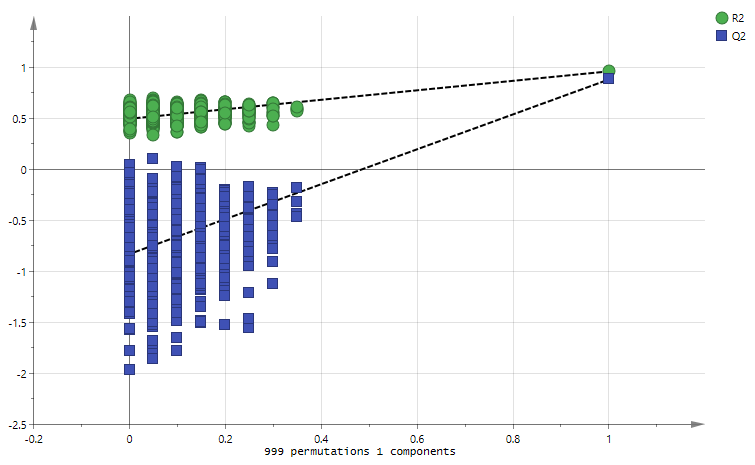  figure S6: Permutations test. The plot shows, for a selected Y-variables, on the vertical axis the values of R2 and Q2 for the original model (far to the right) and of the Y-permuted models further to the left. The horizontal axis shows the correlation between the permuted Y-vectors and the original Y-vector for the selected Y. The original Y has the correlation 1.0 |
| --- |

| 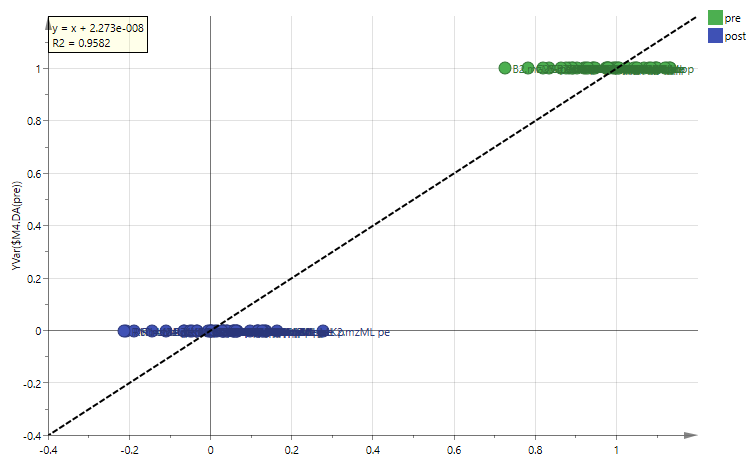  figure S7: The plot displays the observed (y-axis) versus predicted (x-axis) values of the selected Y-variable of the model. The R2 of the regression line indicates the goodness of Fit = 0.95. |
| --- |

| 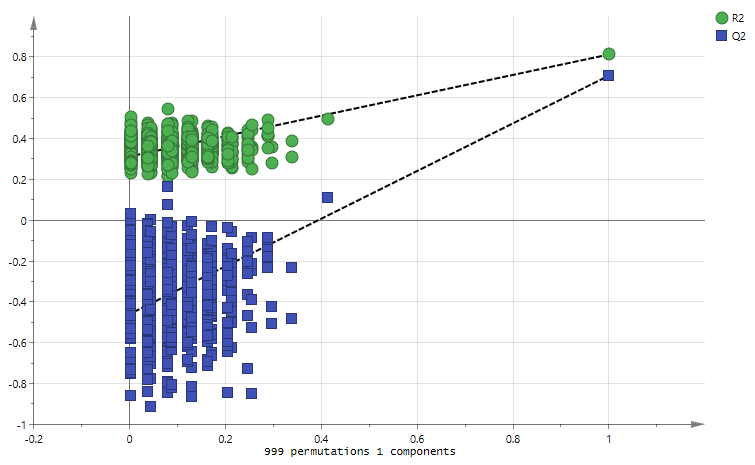  figure S8; Permutations test. The plot shows, for a selected Y-variables, on the vertical axis the values of R2 and Q2 for the original model (far to the right) and of the Y-permuted models further to the left. The horizontal axis shows the correlation between the permuted Y-vectors and the original Y-vector for the selected Y. The original Y has the correlation 1.0 |
| --- |
| 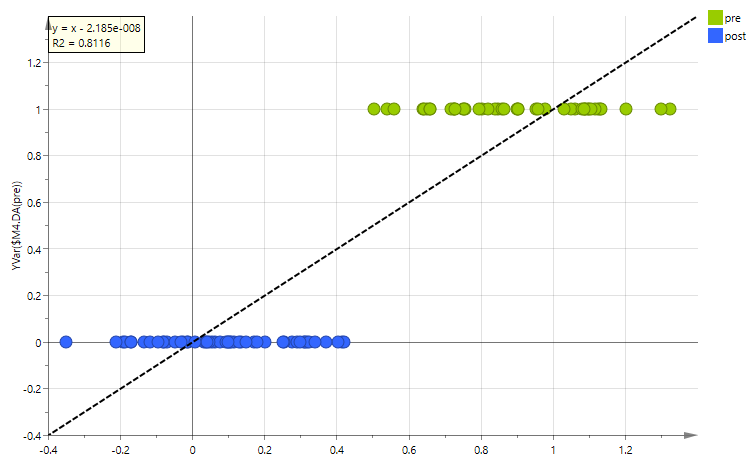  figure S9: The plot displays the observed (y-axis) versus predicted (x-axis) values of the selected Y-variable of the model. The R2 of the regression line indicates the goodness of Fit = 0.81, |

| Mode | m/z | RT (min) | Putative Metabolite | Urine | | Plasma | | Saliva | |
| --- | --- | --- | --- | --- | --- | --- | --- | --- | --- |
|  |  |  |  | **Ratio** | **p-Value** | **Ratio** | **p-Value** | **Ratio** | **p-Value** |
|  |  | **Purine metabolism** | |  |  |  |  |  |  |
| + | 136.061 | 8.7 | Adenine |  |  | 1.354 | 0.015 | 1.314 | 0.032 |
| + | 137.046 | 9.7 | Hypoxanthine * | 0.514 | 0.001 | 0.758 | 0.046 |  |  |
| - | 151.026 | 11.8 | Xanthine |  |  | 1.376 | <0.001 |  |  |
| + | 252.108 | 7.7 | Deoxyadenosine |  |  |  |  | 1.311 | 0.021 |
| + | 253.093 | 8.3 | Deoxyinosine |  |  |  |  | 1.534 | 0.048 |
|  | 298.096 | 6.5 | methylthioadenosine |  |  | 1.481 | 0.003 |  |  |
|  |  | **Arginine and proline metabolism** | |  |  |  |  |  |  |
| - | 104.071 | 13.9 | 4-Aminobutanoate * | 1.335 | 0.059 |  |  |  |  |
| + | 133.097 | 23.5 | Ornithine * |  |  | 1.415 | <0.001 | 1.956 | <0.001 |
| + | 146.092 | 14.9 | 4-Guanidinobutanoate |  |  |  |  | 0.446 | 0.008 |
| + | 146.046 | 15.1 | Glutamate * |  |  | 1.259 | 0.015 |  |  |
| - | 176.103 | 15.9 | L-Citrulline * |  |  | 1.292 | 0.002 |  |  |
| + | 247.14 | 13.9 | N-(Carboxyethyl) arginine |  |  | 1.635 | 0.001 |  |  |
|  |  | **Pyrimidine metabolism** | |  |  |  |  |  |  |
| + | 112.051 | 8.8 | Cytosine |  |  |  |  | 1.406 | 0.02 |
| + | 127.050 | 13.2 | Thymine |  |  | 1.211 | 0.006 | 2.161 | <0.001 |
| + | 243.097 | 12.4 | Thymidine |  |  |  |  | 1.384 | 0.01 |
|  |  |  | **Carnitine metabolism** |  |  |  |  |  |  |
| + | 204.123 | 10.4 | Acetylcarnitine * |  |  | 2.269 | <0.001 |  |  |
| Mode | **m/z** | **RT (min)** | **Putative Metabolite** | **Urine** | | **Plasma** | | **Saliva** | |
|  |  |  |  | **Ratio** | **p-Value** | **Ratio** | **p-Value** | **Ratio** | **p-Value** |
| + | 248.149 | 10.8 | Hydroxybutyrylcarnitine |  |  | 2.209 | <0.001 |  |  |
| + | 274.201 | 6.3 | Heptanoylcarnitine |  |  | 1.692 | 0.003 |  |  |
| + | 302.232 | 5.1 | dimethylheptanoylcarnitine |  |  | 1.988 | 0.001 |  |  |
| + | 312.217 | 5.1 | Decadienoylcarnitine |  |  | 1.101 | 0.032 |  |  |
| + | 314.232 | 4.9 | Decenoylcarnitine * |  |  | 1.632 | 0.001 |  |  |
| + | 330.227 | 5.1 | Keto-decanoylcarnitine |  |  | 1.597 | <0.001 |  |  |
| + | 342.264 | 9.1 | Dodecenoylcarnitine * |  |  | 1.875 | 0.001 |  |  |
| + | 360.274 | 5.1 | 2-Hydroxylauroylcarnitine |  |  | 1.594 | 0.006 |  |  |
| + | 384.270 | 4.8 | Hydroxytetradecadiencarnitie |  |  | 1.658 | <0.001 |  |  |
| + | 386.290 | 4.7 | Hydroxytetradecenoylcarnitie |  |  | 1.700 | 0.001 |  |  |
|  |  | **Pentose phosphate pathway** | |  |  |  |  |  |  |
| - | 151.061 | 12.9 | Xylitol or isomer * |  |  | 1.153 | 0.048 |  |  |
| - | 193.036 | 16.8 | Glucuronate or isomer * |  |  | 1.253 | 0.011 |  |  |
|  |  | Glycine, serine, uratCystiene and threonine metabolism | |  |  |  |  |  |  |
| + | 103.039 | 12.9 | 2-Oxobutanoate |  |  | 0.796 | 0.007 |  |  |
| - | 116.035 | 10.5 | L-2-Amino-3-oxobutanoic acid |  |  | 1.665 | 0.001 |  |  |
| + | 120.065 | 11.3 | Threonine * |  |  | 1.384 | 0.018 |  |  |
|  |  |  | **Lysine metabolism** |  |  |  |  |  |  |
| - | 126.056 | 7.2 | 2,3,4,5-Tetrahydropyridine-2-carboxylate |  |  | 1.271 | 0.034 |  |  |
| + | 146.081 | 14.6 | hexanoic acid |  |  |  |  | 0.465 | 0.017 |
| + | 162.112 | 13.3 | L-Carnitine * |  |  | 2.745 | <0.001 |  |  |
| + | 204.086 | 14.2 | N2-Acetyl-L-aminoadipate |  |  | 1.438 | <0.001 |  |  |
| + | 205.118 | 10.3 | N6-Acetyl-N6-hydroxy-L-lysine |  |  | 1.239 | 0.044 |  |  |
| Mode | **m/z** | **RT (min)** | **Putative Metabolite** | **Urine** | | **Plasma** | | **Saliva** | |
|  |  |  |  | **Ratio** | **p-Value** | **Ratio** | **p-Value** | **Ratio** | **p-Value** |
| + | 219.133 | 14.4 | Carboxyethyllysine |  |  | 1.557 | 0.026 | 1.711 | 0.005 |
|  |  | **Histidine metabolism** | |  |  |  |  |  |  |
| + | 138.043 | 8.8 | Urocanate * |  |  | 1.425 | 0.008 |  |  |
| + | 141.066 | 9.7 | Methylimidazoleacetic acid |  |  | 1.430 | 0.020 |  |  |
| + | 156.076 | 15.4 | L-Histidine | 1.646 | 0.046 |  |  |  |  |
| + | 170.092 | 12.8 | methylhistidine |  |  |  |  | 0.520 | 0.011 |
|  |  | **Tryptophan metabolism** | |  |  |  |  |  |  |
| + | 118.065 | 10.1 | Indole * |  |  |  |  | 1.503 | 0.008 |
| + | 161.107 | 10.1 | Tryptamine |  |  |  |  | 2.021 | 0.002 |
| + | 177.102 | 13.4 | Serotonin |  |  |  |  | 1.580 | 0.002 |
| - | 204.067 | 8.1 | Indolelactate |  |  |  |  | 1.551 | 0.001 |
| - | 219.077 | 8.9 | 5-Hydroxy-L-tryptophan * |  |  |  |  | 2.158 | <0.001 |
|  |  | **Tyrosine metabolism** | |  |  |  |  |  |  |
| - | 179.035 | 8.3 | Hydroxyphenylpyruvate |  |  | 1.424 | 0.002 |  |  |
| - | 181.051 | 9.5 | Hydroxyphenyllactate |  |  | 1.361 | 0.005 |  |  |
| + | 182.081 | 12.3 | L-Tyrosine * |  |  |  |  | 1.449 | 0.002 |
|  |  | **Valine, leucine and isoleucine degradation** | |  |  |  |  |  |  |
| - | 115.040 | 4.8 | 3-Methyl-2-oxobutanoic acid |  |  | 0.765 | 0.005 |  |  |
| - | 129.056 | 4.3 | Methyl-oxopentanoic acid |  |  | 0.718 | 0.001 |  |  |
|  |  | **Phenylalanine metabolism** | |  |  |  |  |  |  |
| + | 122.096 | 4.7 | Phenethylamine |  |  |  |  |  |  |
| + | 123.044 | 13.3 | Benzoate |  |  |  |  | 1.545 | 0.002 |
| + | 136.075 | 13.3 | Phenylacetamide |  |  |  |  | 1.501 | 0.002 |
| Mode | **m/z** | **RT (min)** | **Putative Metabolite** | **Urine** | **Plasma** | **Saliva** | | Mode | |
|  |  |  |  | **Ratio** | **p-Value** | **Ratio** | **p-Value** | **Ratio** | **p-Value** |
| + | 149.059 | 10.1 | Cinnamate |  |  |  |  | 1.394 | 0.020 |
| - | 166.086 | 10.1 | Phenylalanine * |  |  |  |  | 1.537 | 0.004 |
|  |  | **Methionine metabolism** | |  |  |  |  |  |  |
| + | 178.089 | 5.3 | dihomomethionine |  |  | 1.385 | 0.001 |  |  |
|  |  | **Alanine and aspartate metabolism** | |  |  |  |  |  |  |
| + | 161.092 | 11.2 | D-Alanyl-D-alanine |  |  | 0.851 | 0.050 |  |  |
| - | 225.099 | 11.7 | Carnosine * |  |  |  |  | 2.570 | 0.001 |
|  |  | **Fatty acids and metabolites ^C4^** | |  |  |  |  |  |  |
| - | 131.071 | 2.0 | Hydroxyhexanoic acid* ‡ |  |  | 1.213 | 0.038 |  |  |
| - | 227.202 | 15.9 | Tetradecanoic acid ‡ |  |  | 1.763 | 0.008 |  |  |
| - | 241.218 | 17.4 | pentadecanoic acid * ‡ |  |  | 1.243 | 0.004 |  |  |
| - | 253.218 | 16.8 | Hexadecenoic acid ‡ |  |  | 2.158 | 0.002 |  |  |
| - | 255.233 | 18.8 | Hexadecanoic acid isomer ‡ |  |  | 1.465 | 0.001 |  |  |
| - | 281.249 | 19.5 | octadecenoic acid ‡ |  |  | 1.992 | 0.001 |  |  |
|  |  | **Miscellaneous** | |  |  |  |  |  |  |
| + | 160.108 | 14.9 | Guanidinovaleric acid |  |  | 0.649 | 0.002 |  |  |
| - | 259.022 | 15.8 | D-Glucose 1-phosphate * |  |  | 1.41 | 0.002 |  |  |
| + | 345.139 | 12.3 | Melibiitol |  |  | 1.304 | <0.001 |  |  |
| - | 464.302 | 5.5 | Glycocholate * |  |  | 0.337 | 0.003 |  |  |

Table S1. All the metabolites affected significantly by the short-term training trial in the day 2 (p-value < 0.05) or fold change >2 or <0.5. * Matches retention time of standard. ‡ Data from runs on ACE C4 column, otherwise run on the pHILIC column.
